# Pancreatic Cancer after Living-Donor Liver Transplantation: A Case Report of Long-Term Recurrence-Free Survival after Pancreaticoduodenectomy Followed by Adjuvant Chemotherapy

**DOI:** 10.70352/scrj.cr.26-0464

**Published:** 2026-09-26

**Authors:** Naoki Matsuya, Yoshinori Takeda, Shoichi Irie, Takaaki Kato, Ryuji Yoshioka, Yoshihito Kotera, Yoshihiro Mise, Yuki Fukumura, Akio Saiura

**Affiliations:** 1 Department of Hepatobiliary-Pancreatic Surgery, Juntendo University School of Medicine, Juntendo University Hospital, Tokyo, Japan; 2 Department of Human Pathology, Juntendo University School of Medicine, Juntendo University Hospital, Tokyo, Japan

**Keywords:** pancreatic cancer, liver transplantation, pancreaticoduodenectomy, immunosuppression, posttransplant malignancy

## Abstract

**INTRODUCTION:**

Malignancies are a major cause of late mortality after liver transplantation (LT), with an incidence significantly higher than that in the general population. Pancreatic cancer (PC) occurring after LT carries a dismal prognosis; however, the feasibility and outcome of surgery and perioperative chemotherapy remain unclear.

**CASE PRESENTATION:**

A 73-year-old Japanese woman who had undergone living-donor LT 16 years earlier for hepatitis C virus–related cirrhosis was diagnosed with PC during routine CT surveillance in outpatient follow-up. A 23-mm hypovascular tumor in the pancreatic head was found to abut the portal vein (PV), without nodal or distant metastasis. After initiation of neoadjuvant gemcitabine plus S-1, treatment was discontinued during the first cycle because of S-1–induced enteritis requiring hospitalization and intravenous hydration. After recovery of her general condition, she underwent subtotal stomach-preserving pancreaticoduodenectomy with PV resection and reconstruction. During the operation, the pre-existing hepaticojejunostomy was preserved, and pancreaticojejunostomy was created using the blind end of the preserved afferent Roux limb. She was discharged on POD 25 without major complications. Pathological examination revealed well-differentiated pancreatic ductal adenocarcinoma, ypT2N0M0, Stage IB (UICC 9th edition). She subsequently completed 6 months of adjuvant gemcitabine without severe adverse events. No recurrence has been detected during 3 years of postoperative follow-up.

**CONCLUSIONS:**

Early detection and curative-intent surgery followed by adjuvant chemotherapy may contribute to long-term recurrence-free survival in selected LT recipients with localized PC. Careful multidisciplinary management may support safe oncologic treatment in these medically complex patients.

## Abbreviations

ALBI
: albumin–bilirubin
CA19-9
: carbohydrate antigen 19-9
CEA
: carcinoembryonic antigen
CNI
: calcineurin inhibitor
HCV
: hepatitis C virus
LDLT
: living-donor liver transplantation
LT
: liver transplantation
mTOR
: mechanistic target of rapamycin
PC
: pancreatic cancer
PD
: pancreaticoduodenectomy
PV
: portal vein
SIR
: standardized incidence ratio
SMA
: superior mesenteric artery
SMV
: superior mesenteric vein
SSPPD
: subtotal stomach-preserving pancreaticoduodenectomy
TGF-β
: transforming growth factor-β
UICC
: Union for International Cancer Control

## INTRODUCTION

Over the past 30 years, advances in immunosuppressive agents, surgical techniques, and postoperative management have led to substantial improvements in short-term outcomes of organ transplantation. Although LT has also achieved remarkable gains in long-term outcomes, with the 1-year survival rate now exceeding 90%,^1)^ improvement in long-term survival has plateaued over the past several decades, and malignancy remains the leading cause of death.^2)^ Malignancies are more common in LT recipients than in the general population.^3,4)^ This may be partly attributable to CNI-based immunosuppression, which remains the backbone of posttransplant therapy,^5)^ through pathways mediated by TGF-β.^6)^

PC remains one of the leading causes of cancer-related mortality worldwide.^7,8)^ Its risk is increased after LT (SIR, 1.65).^9)^ Standard treatment for PC consists of surgical resection combined with perioperative chemotherapy.^10–12)^ However, LT recipients may be poor candidates for these therapies because surgery can be technically demanding owing to postoperative anatomical complexity, and chemotherapy may cause severe adverse events in the setting of ongoing immunosuppressive treatment. Despite the increased risk of malignancy in LT patients, few studies have evaluated the safety and efficacy of contemporary treatment strategies for PC in LT recipients. Here, we report a patient who underwent PD and perioperative chemotherapy and completed 3 years of postoperative follow-up without recurrence.

### CASE PRESENTATION

A 73-year-old Japanese woman had undergone LDLT 16 years earlier for HCV-related cirrhosis and had since been followed regularly at our institution. A left-lobe graft including the middle hepatic vein but without the caudate lobe was transplanted, and biliary reconstruction was performed using a retrocolic Roux-en-Y hepaticojejunostomy to the graft hepatic duct. She was maintained on tacrolimus monotherapy at 2 mg/day for immunosuppression. Her medical history included hypertension and type 2 diabetes mellitus, neither of which required pharmacologic treatment.

Although she remained asymptomatic, routine contrast-enhanced CT revealed a 23-mm hypovascular tumor in the pancreatic head, accompanied by dilatation of the distal main pancreatic duct (**Fig. 1A**). Endoscopic US-guided fine-needle aspiration confirmed adenocarcinoma. The tumor was in contact with the PV over approximately 60 degrees of its circumference, without vessel stenosis or contour irregularity (**Fig. 1B**). No regional lymphadenopathy or distant metastases were detected.

**Fig. 1:**
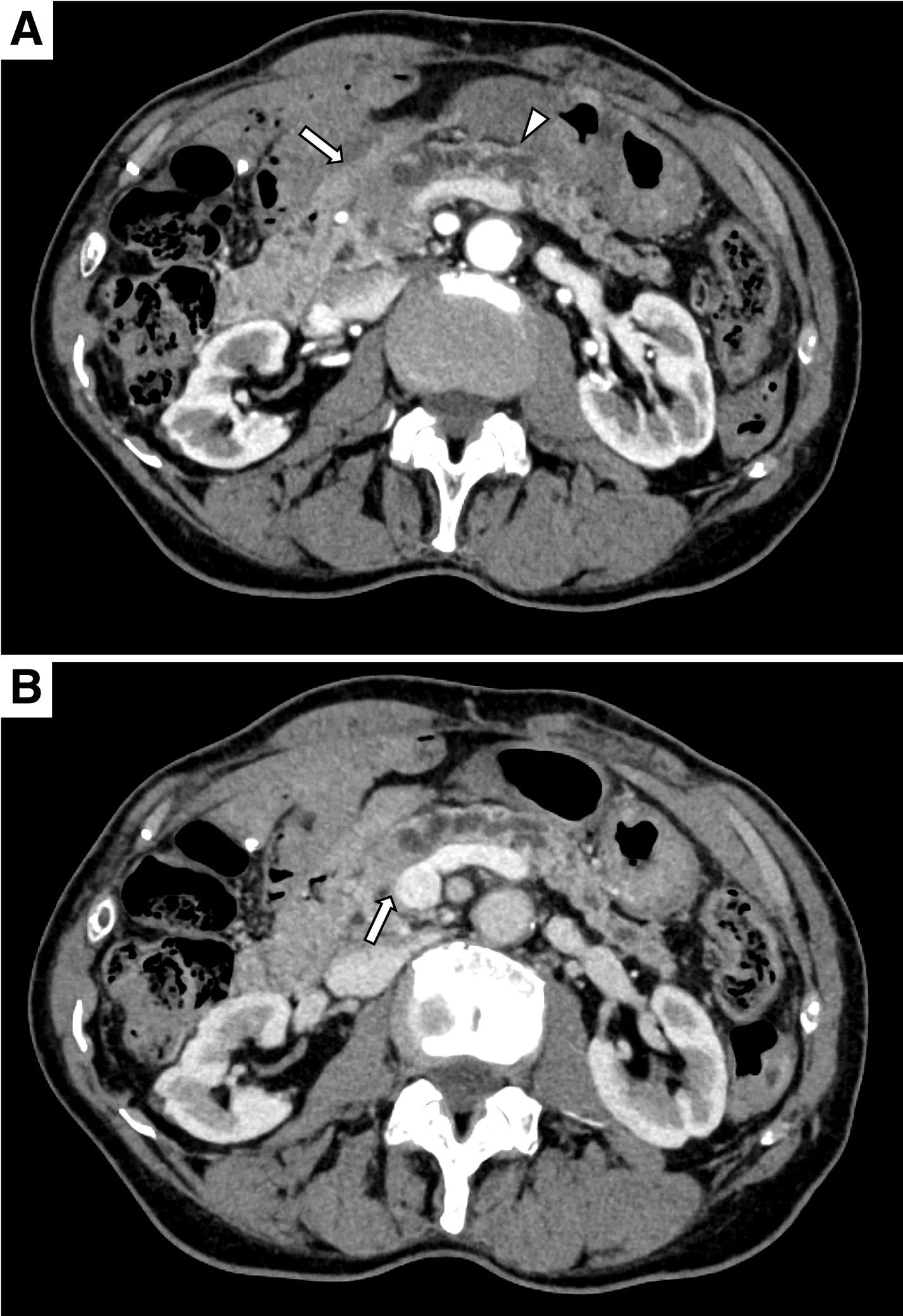
Contrast-enhanced CT scan at initial presentation. A 23-mm hypovascular tumor was detected in the pancreatic head on contrast-enhanced CT imaging. (**A**) Arterial phase showed subtle early enhancement of the lesion (white arrow) with associated mild distal pancreatic ductal dilatation (arrowhead). (**B**) Portal venous phase demonstrated the tumor abutting the PV over approximately 60 degrees of its circumference, without evidence of stenosis or contour irregularity (white arrow). PV, portal vein

Laboratory tests showed preserved liver graft function, with a total bilirubin level of 0.55 mg/dL, serum albumin level of 3.9 g/dL, and prothrombin time-international normalized ratio of 1.01. The patient had neither ascites nor hepatic encephalopathy. The Child–Pugh score was 5 (class A), and the ALBI score was −2.67 (grade 1). Tumor markers were within or near the normal range: CA19-9, 19 U/mL; CEA, 5.1 ng/mL. Accordingly, she was diagnosed with resectable pancreatic head cancer, cT2N0M0, Stage IB (UICC 9th edition).

Neoadjuvant chemotherapy with gemcitabine plus S-1 was initiated using the planned full-dose regimen: gemcitabine 1000 mg/m^2^ on days 1 and 8 plus oral S-1 100 mg/day on days 1–14 of a 21-day cycle.^12)^ However, treatment was discontinued during the first cycle because of S-1–induced enteritis with grade 3 diarrhea according to the Common Terminology Criteria for Adverse Events, requiring hospitalization and intravenous hydration. After her general condition improved, she underwent SSPPD with en bloc segmental resection of the PV–SMV confluence 18 days after discontinuation of neoadjuvant chemotherapy.

Intraoperative exploration revealed dense adhesions around the hepatic hilum and liver graft (**Fig. 2A**). A schematic overview of the preoperative post-LDLT anatomy is shown in **Fig. 3A** and **3B**, including the pre-existing retrocolic Roux-en-Y hepaticojejunostomy and jejunojejunostomy, the pancreatic head tumor abutting the PV, the previous graft hepatic arterial and portal venous anastomoses, and the operative field for pancreatic resection and regional lymphadenectomy. At surgery, an anterior approach to the SMA was used. The first and second portions of the pancreatic head nerve plexus (PLph I and PLph II) were dissected, while the nerve plexus around the SMA (PLsma) was preserved. Standard regional lymphadenectomy for pancreatic head cancer was performed, including dissection of lymph node stations nos. 5, 6, 8, 12, 13, 14, and 17, as well as lymph nodes in the proximal jejunal mesentery. However, lymphadenectomy within the hepatoduodenal ligament was limited to the area caudal to the hepatic arterial and portal venous anastomoses created during LT to avoid injury to these vascular anastomoses. The pre-existing Roux limb used for hepaticojejunostomy was identified and carefully mobilized from the undersurface of the graft and from the transverse colon and mesocolon. The pre-existing jejunojejunostomy was located approximately 30 cm distal to the ligament of Treitz. The jejunum was divided proximal to this anastomosis, as indicated in **Fig. 3A**. The distal jejunal stump, which remained continuous with the preserved pre-existing jejunojejunostomy, was subsequently used for gastrojejunostomy. A preoperative review of the operative records and imaging studies indicated that the blind end of the elevated Roux limb was sufficiently long. Intraoperatively, the distance between the pre-existing hepaticojejunostomy and the blind end was confirmed to be approximately 25 cm. After adequate mobilization without division of the mesentery, the blind end reached the pancreatic stump without tension and was therefore used for pancreaticojejunostomy. The pre-existing hepaticojejunostomy was left intact. PV resection was performed caudal to the previous PV anastomosis created during LDLT; therefore, the previous PV anastomosis was preserved. The PV–SMV continuity was reconstructed by direct end-to-end anastomosis (**Fig. 2B**). Pancreaticojejunostomy was created using the modified Kakita technique with duct-to-mucosa anastomosis and an external pancreatic duct stent. The final arrangement consisted of the preserved hepaticojejunostomy, the newly created pancreaticojejunostomy at the blind end of the Roux limb, and gastrojejunostomy, as illustrated in **Fig. 3C**.

**Fig. 2:**
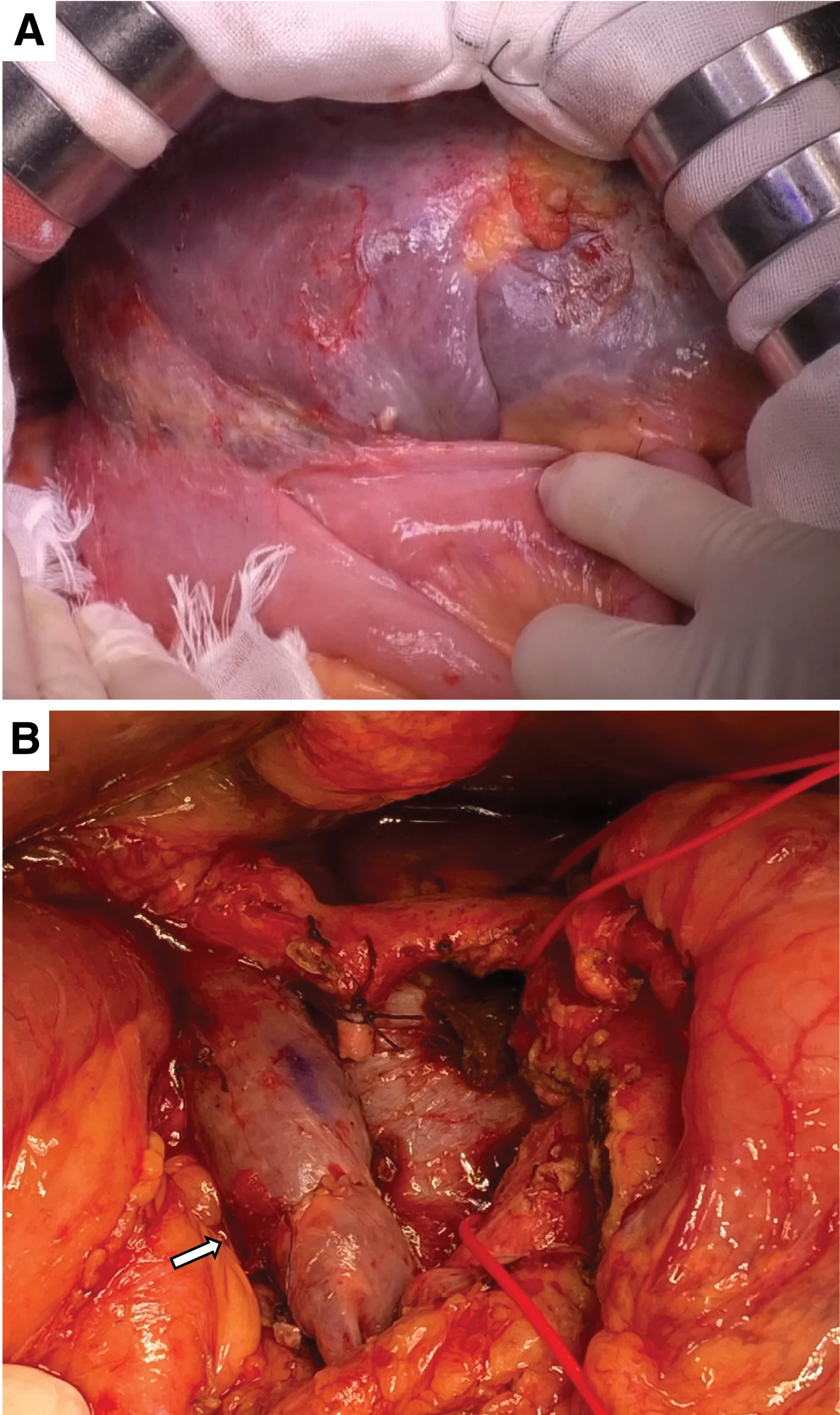
Intraoperative photographs. (**A**) Intraoperative findings at laparotomy demonstrating severe adhesions surrounding the transplanted liver. (**B**) Operative view after specimen removal, showing the reconstructed PV (white arrow). PV, portal vein

**Fig. 3:**
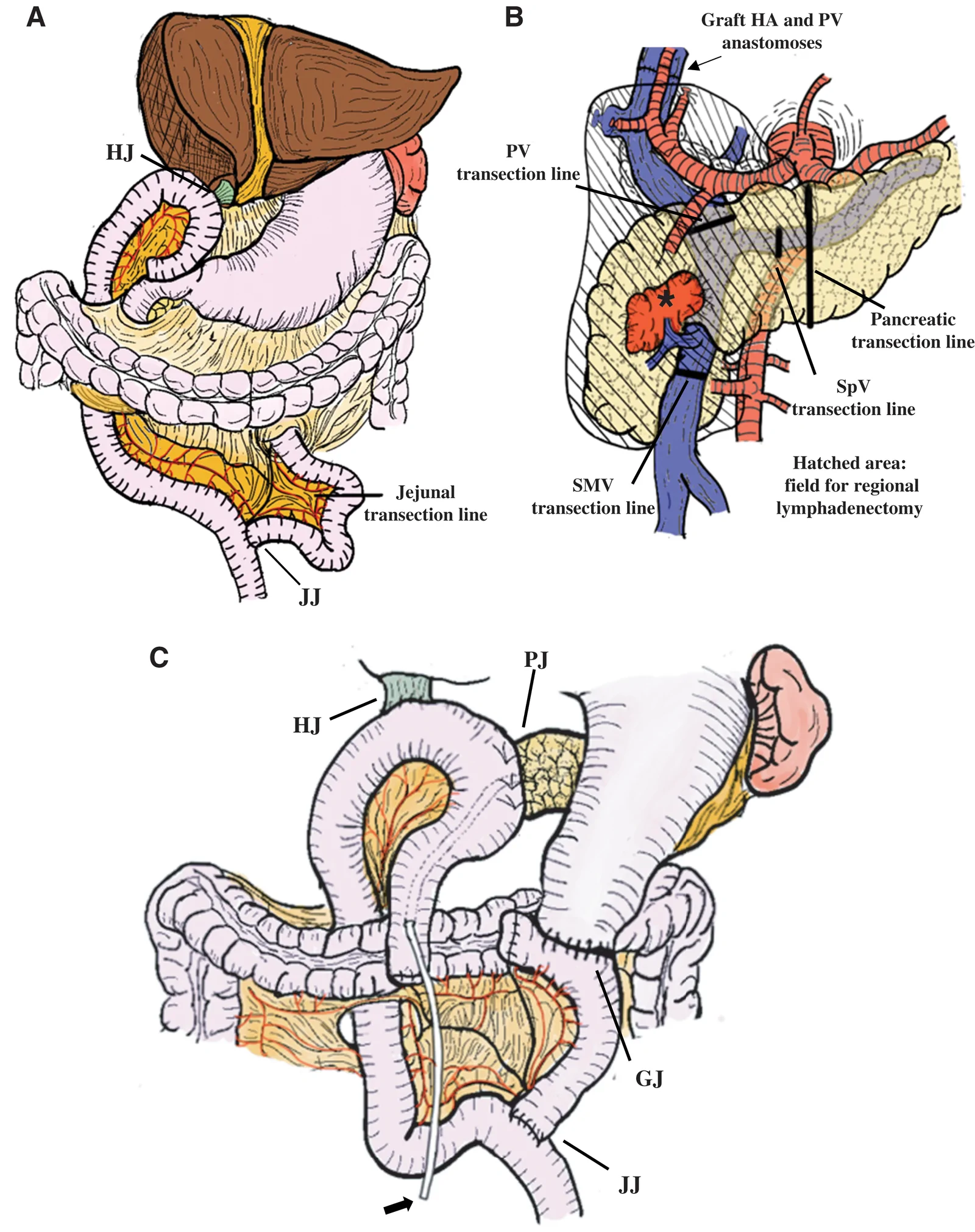
Schematic illustrations of the preoperative anatomy after LDLT and the final reconstruction after SSPPD. (**A**) Pre-existing retrocolic Roux-en-Y reconstruction after LDLT, showing the HJ to the graft hepatic duct and the downstream JJ. The transverse black line indicates the jejunal transection site proximal to the pre-existing JJ. (**B**) Close-up of the pancreatic head before PD, showing the tumor (*) in contact with the PV. The graft HA and PV anastomoses created during LDLT are also shown. The bold lines show the pancreatic and PV transection lines. The hatched area indicates the field for regional lymphadenectomy. Within the hepatoduodenal ligament, the cranial limit of lymphadenectomy was the bifurcation of the hepatic artery into the right and left hepatic arteries. (**C**) Final reconstruction after SSPPD with resection of the PV–SMV confluence. The pre-existing HJ and JJ were preserved. A PJ was created at the blind end of the afferent Roux limb, followed by GJ. The black arrow indicates the external pancreatic duct stent. GJ, gastrojejunostomy; HA, hepatic artery; HJ, hepaticojejunostomy; JJ, jejunojejunostomy; LDLT, living-donor liver transplantation; PD, pancreaticoduodenectomy; PJ, pancreaticojejunostomy; PV, portal vein; SMV, superior mesenteric vein; SSPPD, subtotal stomach-preserving pancreaticoduodenectomy

The operation time was 438 minutes, and the estimated blood loss was 135 mL. No intraoperative complications occurred. The patient received prophylactic antibiotics for the first 2 PODs. Because laboratory tests showed elevated inflammatory markers, antibiotic therapy was switched to piperacillin/tazobactam, which was administered from PODs 3 to 7. She developed transient delayed gastric emptying and neurogenic diarrhea, both of which were managed conservatively. No infectious or anastomotic complications were observed. She was discharged on POD 25.

Histopathological examination revealed invasive ductal adenocarcinoma, predominantly well-differentiated with a minor moderately differentiated component (**Fig. 4**). The pathological tumor diameter was 23 × 18 × 18 mm, and the histological response to neoadjuvant chemotherapy was Grade 1a according to the Japan Pancreas Society criteria. No lymph node metastasis was identified among 34 examined lymph nodes. Microscopic invasion of the resected PV wall was absent. Lymphatic and venous invasion were absent, whereas perineural invasion was present. All resection margins were negative (R0), and the pathological stage was ypT2N0M0, Stage IB (UICC 9th edition). Given the severe S-1–induced enteritis that occurred during neoadjuvant therapy, the multidisciplinary team decided to administer dose-reduced adjuvant gemcitabine, which she completed for 6 months at 80% of the planned dose. During the perioperative period, tacrolimus monotherapy was continued at 2 mg/day with therapeutic drug monitoring, and trough levels ranged from 3.0 to 5.4 ng/mL. During adjuvant chemotherapy, tacrolimus monotherapy was also continued at 2 mg/day with ongoing therapeutic drug monitoring, and trough levels ranged from 3.1 to 5.5 ng/mL. Liver graft function remained stable, with no clinical or biochemical evidence of rejection, and no severe infectious complications occurred. No recurrence has been detected during 3 years of postoperative imaging surveillance.

**Fig. 4:**
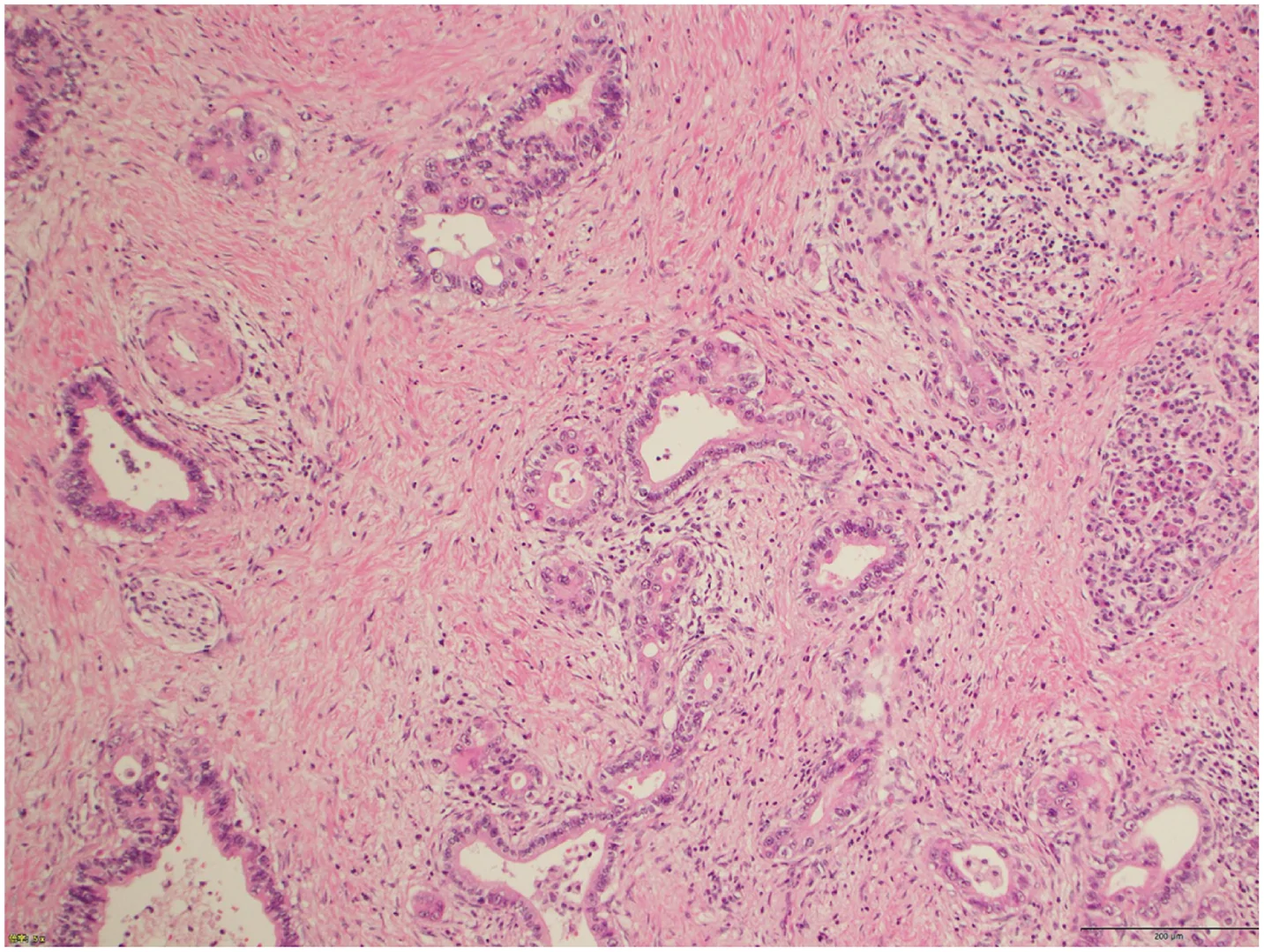
Histopathological findings of the resected specimen (hematoxylin and eosin stain). The tumor was diagnosed as invasive ductal adenocarcinoma, predominantly well differentiated, following neoadjuvant chemotherapy. Atypical glandular and papillary structures infiltrate the dense desmoplastic stroma.

## DISCUSSION

We report a case of pancreatic head adenocarcinoma diagnosed 16 years after LDLT. The asymptomatic tumor was detected at a localized stage on routine imaging surveillance. The patient underwent SSPPD with PV resection and completed 6 months of adjuvant gemcitabine, and no recurrence has been detected during 3 years of postoperative follow-up. Among previously published reports, the present case appears to represent the longest documented recurrence-free survival after curative-intent resection for pancreatic ductal adenocarcinoma following LT. This case suggests that careful standard multidisciplinary therapy for PC may be feasible in selected patients.

De novo malignancy is an important cause of late mortality after LT. In a large registry analysis of 1-year LT survivors, malignancy was the most common cause of death, accounting for 16.4% of long-term deaths, and its contribution increased from 15% in 1987–1990 to 27% in 2011–2016.^2)^ In a multicenter cohort, de novo malignancy developed in 22% of LT recipients within 12 years, with 10-year cumulative incidences of 10.8% for skin malignancy, 10.5% for solid-organ malignancy, and 3.2% for hematologic malignancy.^3)^ Regarding organ-specific patterns, gastrointestinal, lung, genitourinary, and oropharyngeal/laryngeal cancers are relatively common among non-skin malignancies after LT.^3,5)^ Although PC is rare among post-LT malignancies, its risk appears to be increased compared with the general population (SIR, 1.65).^9)^ In that registry-based study, nearly half of patients were diagnosed with PC at a local or regional stage; nevertheless, the median overall survival after diagnosis was approximately 4 months, highlighting the difficulty of treating PC after LT.^9)^ Previous case series have described the clinical courses of patients with PC after LT who were treated with surgery and perioperative chemotherapy (**Table 1**). However, the feasibility and outcomes of such treatment in this population have not yet been established.^13–16)^ Our detailed report may therefore serve as a practical reference for the management of PC in these complex and medically vulnerable post-LT patients.

**Table 1:** Previous case reports and case series of PC in liver transplant recipients and the present case

Year/author	Age/sex	Indication for transplantation	Time from LT to PC diagnosis (years)	Immunosuppressive therapy	Tumor location, TNM classification	Treatment	Chemotherapy regimen	Recurrence/progression	Survival/status
2009 Stauffer	56/M	Alpha-1 antitrypsin deficiency	3.8	Tacrolimus, MMF, steroids	Ph; size NR; T3N1	Resection	NR	Recurrence (site NR)	21 mo; DOD
2010 Sutcliffe	40/M	PSC	3	Sirolimus-based after PD	Ph; 3 cm; T3N1	Resection	Adjuvant gemcitabine	Locoregional recurrence	10 mo; DOD
2014 Mejia	Series (n = 4)	Various/NR	NR	Tacrolimus-based protocol	Ph; size NR; T3N1 (n = 3); T3N0 (n = 1)	Resection	NR	NR	Median 12 mo; status NR
2021 Rauf	71/M	HBV, hereditary hemochromatosis	16	CNI, steroids, MMF	Ph; 3.2 cm; M1 intraoperatively	Resection	Adjuvant chemotherapy; regimen NR	Progression	12 mo; DOD
	74/F	Alcoholic liver disease	18	CNI, steroids, MMF	Ph; 4.0 cm; M1	Palliative care	None	Progression	5 mo; DOD
	66/M	HCV (HCC on explant)	9	CNI, steroids, MMF	Ph; 3.0 cm; SMA invasion	Palliative care	None	Progression	9 mo; DOD
	48/M	HCV, alcoholic liver disease	3	CNI, steroids, MMF	Ph; 3.1 cm	Resection	NR	Distant recurrence	4 mo; DOD
	66/F	HCV (iCCA on explant)	8	CNI, steroids, MMF	Ph; 5.3 cm; SMA, SMV, and celiac axis invasion	Palliative chemotherapy	Gemcitabine + nab-paclitaxel	Progression	6 mo; DOD
	61/M	NASH, HCC	5	CNI, steroids, MMF	Ph; size NR	Resection	NR	Distant recurrence	17 mo; DOD
2026 Ours	73/F	HCV-related cirrhosis	16	Tacrolimus monotherapy	Ph; 23 mm; resectable; ypT2N0M0, Stage IB	Resection	NAC GS (discontinued); Adjuvant gemcitabine	None	40 mo; NED

Data are shown as reported in the original articles. NR indicates not reported.

CNI, calcineurin inhibitor; DOD, died of disease; F, female; GS, gemcitabine plus S-1; HBV, hepatitis B virus; HCC, hepatocellular carcinoma; HCV, hepatitis C virus; iCCA, intrahepatic cholangiocarcinoma; LT, liver transplantation; M, male; M1, distant metastasis; MMF, mycophenolate mofetil; NAC, neoadjuvant chemotherapy; NASH, nonalcoholic steatohepatitis; NED, no evidence of disease; NR, not reported; PC, pancreatic cancer; PD, pancreaticoduodenectomy; Ph, pancreatic head; PSC, primary sclerosing cholangitis; SMA, superior mesenteric artery; SMV, superior mesenteric vein; TNM, tumor–node–metastasis

Although curative-intent surgery remains essential for long-term survival in patients with PC, undergoing PD after LT is technically challenging for several reasons. First, dense adhesions and posttransplant anatomical distortion often obscure normal tissue planes, which may lead to injury of critical organs or vessels. In particular, preservation of the blood supply to the afferent Roux limb is crucial, as ischemic injury to the afferent limb may jeopardize the pre-existing hepaticojejunostomy; therefore, meticulous adhesiolysis is required. In this case, a precise understanding of the posttransplant anatomy based on the operative record of the prior LT, together with careful preoperative evaluation using CT imaging, helped identify altered vascular and biliary anatomy, including the course of the afferent limb and the hepaticojejunostomy, and anticipate structures that should be preserved during surgery. During surgery, early identification of the afferent Roux limb and the inferior vena cava also contributed to procedural safety, as these structures served as key landmarks for identifying adjacent structures.

Second, reconstructive strategies for PD after LT are not standardized because the preserved anatomy varies among patients; therefore, careful preoperative planning and intraoperative decision-making are essential. Sugiura et al. proposed a flowchart for selecting the method of reconstruction after PD following surgery for perihilar cholangiocarcinoma, according to whether the pre-existing hepaticojejunostomy can be preserved and whether the afferent limb is available.^17)^ This approach may provide a useful conceptual framework for planning reconstruction in patients after LT. In the present case, the hepaticojejunostomy could be preserved, and the blind end of the afferent limb reached the pancreatic stump without tension; therefore, Child-type reconstruction was selected as the most straightforward reconstructive option in this anatomical setting. When the afferent limb is not available, alternative reconstructive strategies, including double-tract reconstruction, should be considered. Because reconstruction must be individualized according to preserved anatomy and intraoperative findings, careful preoperative planning with anticipation of multiple reconstructive scenarios is essential.

Previous reports suggested that infectious complications may occur more frequently in transplant recipients because of chronic immunosuppression.^16,18,19)^ Mejia et al. reported that 5 of 18 patients developed infectious complications after pancreatic surgery following LT.^16)^ Moreover, Blake et al. reported that 1 of 9 patients who underwent PD after LT died within 90 days due to sepsis caused by an ischemic Roux-en-Y limb.^18)^ De’Angelis et al. emphasized that abdominal surgery in solid organ transplant recipients carries substantial morbidity and that infectious complications are a major concern under chronic immunosuppression; they also noted that atypical presentations may delay the diagnosis of infection.^19)^ As optimal perioperative management strategies, such as the appropriate duration of antibiotic prophylaxis, have not been established for pancreatic surgery after LT, we treated this patient according to standard perioperative management principles used for non-transplant patients with particularly careful postoperative surveillance.

Successful completion of adjuvant chemotherapy may also have contributed to the long-term recurrence-free survival. Establishing a safe systemic chemotherapy regimen for LT recipients remains challenging because standardized protocols are lacking and treatment-related toxicities, particularly infectious complications, may be amplified by concomitant immunosuppression.^20)^ In addition, clinically relevant drug–drug interactions involving CNI require careful attention.^21–23)^ To mitigate these risks, cytotoxic chemotherapy should be delivered with individualized immunosuppression and close multidisciplinary monitoring, including vigilant surveillance for infection, as well as careful assessment of graft function, renal function, and tacrolimus trough levels.^21–23)^ In the present case, adjuvant gemcitabine was completed without graft rejection or severe infectious complications. This experience suggests that, with careful management, systemic therapy may be feasible after LT and may offer oncologic benefit in appropriately selected patients.

In this patient, tacrolimus was maintained throughout the perioperative period because perioperative initiation of an mTOR inhibitor may impair wound and anastomotic healing.^21,24)^ Although mTOR inhibitor–based immunosuppression has been reported to reduce the risk of recurrence of certain malignancies after transplantation, postoperative conversion was not undertaken in this patient because evidence supporting its oncological benefit in de novo PC after LT remains limited, and stable graft function had been maintained with low-dose tacrolimus monotherapy of 2 mg/day (trough level approximately 5 ng/mL).^25)^

Early detection is another key determinant of favorable survival in PC, as in the non-LT setting.^26,27)^ Rauf et al. noted that the diagnosis of PC in patients after LT can be challenging because jaundice is often attributed to benign biliary strictures or graft dysfunction.^13)^ Moreover, as demonstrated in the present case, patients who have undergone Roux-en-Y hepaticojejunostomy may not develop jaundice due to altered post-LT anatomy. Therefore, periodic imaging may be a reasonable approach to facilitate the detection of early-stage tumors after LT. In support of this approach, Uemura et al. demonstrated that routine imaging increased the likelihood of detecting malignancies at a resectable stage and achieving complete resection in LT recipients.^28)^ Given the increased risk of malignancy after LT, structured screening strategies warrant further investigation.

## CONCLUSIONS

Following surgery and adjuvant chemotherapy, the patient remained recurrence-free for 3 years. Although the favorable outcome observed in this single case should be interpreted cautiously, surgical resection followed by adjuvant chemotherapy may represent a treatment option for carefully selected patients with PC after LT.

## DECLARATIONS

### Ethics statement on organ source

The liver graft used in this case was obtained from a living donor. Autonomous consent free from coercion was obtained from both the donor and the recipient. The organ was not sourced from executed prisoners or prisoners of conscience.

### Funding 

None.

### Authors’ contributions 

NM collected the patient data and drafted the manuscript.

YT and AS conceived the surgical strategy, performed the surgery, supervised the patient’s treatment, and critically revised the manuscript for important intellectual content.

YF made the pathological diagnosis.

SI, TK, RY, YK, and YM critically reviewed and revised the manuscript for important intellectual content.

All authors read and approved the final manuscript and agree to be accountable for all aspects of the work.

### Availability of data and materials 

The data are available upon a reasonable request.

### Ethics approval and consent to participate 

Ethical approval was not required for this case report according to our institution’s policy.

### Consent for publication 

Written informed consent was obtained from the patient for publication of this case report and any accompanying images.

### Competing Interests

The authors declare that they have no competing interests.
